# Performance of genetic programming optimised Bowtie2 on genome comparison and analytic testing (GCAT) benchmarks

**DOI:** 10.1186/s13040-014-0034-0

**Published:** 2015-01-08

**Authors:** W B Langdon

**Affiliations:** Department of Computer ScienceUniversity College London, Gower Street, London, WC1E 6BT UK

**Keywords:** Double-ended DNA sequence, High throughput Solexa 454 nextgen NGS sequence query, Rapid fuzzy string matching, Homo sapiens genome reference consortium HG19

## Abstract

**Background:**

Genetic studies are increasingly based on short noisy next generation scanners. Typically complete DNA sequences are assembled by matching short NextGen sequences against reference genomes. Despite considerable algorithmic gains since the turn of the millennium, matching both single ended and paired end strings to a reference remains computationally demanding. Further tailoring Bioinformatics tools to each new task or scanner remains highly skilled and labour intensive. With this in mind, we recently demonstrated a genetic programming based automated technique which generated a version of the state-of-the-art alignment tool Bowtie2 which was considerably faster on short sequences produced by a scanner at the Broad Institute and released as part of The Thousand Genome Project.

**Results:**

Bowtie2 ^*GP*^ and the original Bowtie2 release were compared on bioplanet’s GCAT synthetic benchmarks. Bowtie2 ^*GP*^ enhancements were also applied to the latest Bowtie2 release (2.2.3, 29 May 2014) and retained both the GP and the manually introduced improvements.

**Conclusions:**

On both singled ended and paired-end synthetic next generation DNA sequence GCAT benchmarks Bowtie2GP runs up to 45% faster than Bowtie2. The lost in accuracy can be as little as 0.2–0.5% but up to 2.5% for longer sequences.

## Background

“Next generation sequencing (NGS) technology has become the *de facto* indispensable tool to study genomics and epigenomics in recent years” [1]. Although NGS DNA scanners can generate in the region of a billion DNA sequences per run, each sequence is only a few hundred base pairs (bp) long [2]. Typically each sequence is aligned against an existing reference genome. While in many cases DNA sequences match the reference exactly, since next generation scanners are inherently noisy, it is common practise to scan the target sample three times. Consistent differences between NGS sequences and the reference genome may indicate mutations in the sample. The 1000 Genomes Project used next generation scanners to identify 15 million single base changes (SNPs) and more complex mutations [3,4].

Many genomes contain repeated DNA sequences [5], thus a short DNA sequence may match a reference genome more than once. To extend the usefulness of existing scanners, they can be used to generate “paired-end” sequences, in which both ends of longer DNA strands are sequenced in the normal way but the connecting part is not [6]. The length of the unsequenced region is known only approximately. Typically it is in the region of 500 bp. If one end lies in a repeated sequence, the other end can be used. However even if both ends lie in repeated sequences, typically knowing approximately how far apart the two ends are is sufficient to remove the ambiguity of both ends matching multiple times.

Matching (also known as mapping) biological sequences is essentially a computational task. Indeed it remains the life blood of Bioinformatics. BLAST [7] remains the gold standard computer program for approximate biological string matching. However it is usually considered far too slow to use with the huge volume of data generated by NextGen scanners. Bowtie [8] was the first fast program able to deal with NextGen DNA sequences and able to compress the human genome into less than 4 GBytes. (Fitting into four gigabytes enabled Bowtie to run on typical desktop computers then available.) However Bowtie is limited in the types of mutation it can deal with and so it was re-written to give the more functional Bowtie2 [9]. Perhaps surprisingly some of the best programs are publicly funded open source and freely available rather than commercial. E.g. BWA [10] and Bowtie2.

### Introduction

Genetic programming (GP) [11,12] is one of a family of computer techniques [13-15] which use Darwin’s theory of evolution of species by natural selection [16] by applying survival of the fitness to an artificial population inside the computer. Typically the programmer provides an external fitness function which is used to determine the fitness of individuals in the population and so who survives and has children. Children are created by applying operations analogous to mutation and recombination to their parents. In the case of GP the population contains a species of computer programs. Special mutation and recombination operations are used which ensure the children are syntactically correct programs and their fitness is calculated by running them on input data from the problem and assessing the quality of their answers.

In the http://www.cs.ucl.ac.uk/staff/W.Langdon/gismo/ Gismo project instead of evolving complete programs, we used GP to evolve a population of patches to Bowtie2. GP was used in combination with other search based software engineering techniques [17] to automatically tailor Bowtie2, giving a version which runs considerably faster than the original released code on “single ended” short (36 bp) DNA sequences produced by the Broad Institute’s Illumina Genome Analyzer II Solexa scanner. The goal was to find matches in the human genome faster without unduly sacrificing the quality of the matches. On out-of-sample Solexa sequences on average it runs more than 70 times faster than the original release of Bowtie2 and finds very slightly better matches [18].

While we would normally advocate re-optimising the Bowtie2 C++ code for new circumstances, we show the optimised version can also process DNA sequences from other sources.

On paired-end data from the UCL http://www.ucl.ac.uk/cancer/ Cancer Institute human blood studies we found Bowtie2 ^*GP*^ not only ran faster than Bowtie2 but was also more than four times faster than the Bioinformatics sequencing tool (BWA [10]) currently used by the Cancer Institute, takes less memory and yet finds similar matches in the human genome [19].

Next we submitted 4 versions of Bowtie2 to the widely respected GCAT [20] synthetic NextGen benchmarks (the original Bowtie2 and the current release and GP updated versions of both). Unlike the real data supplied by the Cancer Institute, GCAT has the advantage that the sequences have been prepared against a reference genome and so have both defined noise characteristics and known ground truth. Since the correct mapping is known, GCAT can readily calculate the fraction which are correct.

## Method

The DNA test sequences for eight GCAT benchmarks were down loaded via the GCAT eb interface at http://www.bioplanet.com/gcat/ in fastq format. The benchmarks cover sequences of lengths 100 base pairs, 150 bp, 250 bp and 400 bp, for both paired end and single ended small-indel tests. There are two files for each of the paired end and one _1.fastq file for each single ended benchmarks. Making a total of 41 509 741 sequences, occupying 25 gigabytes.

As recommended by GCAT, pre-built Bowtie2 index files for the human genome (hg19, GRCh37 Genome Reference Consortium Human Reference 37 (GCA_000001405.1)) were down loaded from ftp://ftp.ccb.jhu.edu/pub/data/bowtie2_indexes/hg19.zip Decompressed the.bt2 files occupy 4 GB in total.

As recommended by GCAT, samtools was used to convert Bowtie2 sam output to the bam format used by GCAT. Pre built 64-bit executable programs for Linux samtools were obtained from SourceForge (http://sourceforge.net/projects/samtools/files/samtools/1.0/

We used four versions of Bowtie2; two from Ben Langmead and two after GP improvement. The original release of Bowtie2 (version 2.0.0 beta, 16 Oct 2011) has been updated automatically by genetic programming to give Bowtie2 ^*GP*^ [18]. (Bowtie2 ^*GP*^ is available via http://www.cs.ucl.ac.uk/staff/W.Langdon/ftp/gp-code/bowtie2gp W.Langdon/ftp/gp-code/bowtie2gp.) The seven changes made by genetic programming are given in ([18] Figure fifteen). Bowtie2 has been changed by Langmead’s team many times since 2011. (GitHub lists more than 700). In particular, possibly unwittingly, they have applied the first three optimisations found by GP to version 2.2.3 (30 May 2014). The sources of Bowtie2 2.2.3 were downloaded from SourceForge http://sourceforge.net/projects/bowtie-bio/files/bowtie2/2.2.3/bowtie2-2.2.3-source.zip/download.

Firstly Bowtie2 2.2.3 was built for 64-bit Linux. Then the remaining four GP changes were applied to the 2.2.3 source code and Bowtie2 ^*GP*^ was built from them. (Bowtie2 ^*GP*^ 2.2.3 can be obtained via anonymous FTP or http://www.cs.ucl.ac.uk/staff/W.Langdon/ftp/gp-code/bowtie2gp/bowtie2-2.2.3gp-align-s. In all four cases the gcc 4.1.2 compiler optimisation etc. used to build the programs were those used by the build process supplied with the corresponding Bowtie2 sources.

### Command lines

Essentially we used the same command line as GCAT itself used when they ran Bowtie2. E.g. bowtie2gp –seed 133540 -I 450 -X 550 –sensitive -x hg19 -1 gcat_set_037_1.fastq -2 gcat_set_037_2.fastq Bowtie2 uses pseudo random numbers internally. Where multiple runs are made to estimate variability up to five different –seed values were used. With paired-end sequences the -I and -X parameters give the range of separation allowed between the two ends. (As mentioned above, GCAT uses synthetic data, and the actual separation is known to be 500, nevertheless, as recommend by GCAT, we used the same -I and -X as had been used by GCAT when they ran Bowtie2 internally). Again –sensitive was recommended by GCAT. Although Bowtie2 supports multiple threads to take advantage of multicore architectures, to allow ease of comparison only a single server CPU core was used. All runs were made on the same 32 GB eight 3.00 GHz core server. Finally (not shown), also recommended by GCAT, we used the unix command /usr/bin/time -v to gather performance information.

samtools view -b was used to convert Bowtie2’s output to bam format. Typically it takes samtools about two minutes to convert Bowtie2’s output of 3GB to a compressed binary bam file of 700MB.

## Results and discussion

**Figure 1:**
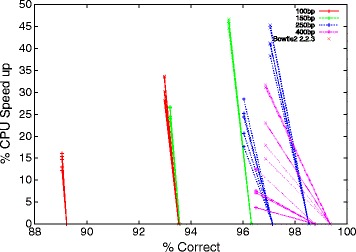
**Speed up of genetic programming versions of Bowtie2 compared to hand produced code on single ended small-indel GCAT benchmarks.** The horizontal axis gives the fraction of sequences correctly mapped (given by GCAT itself). The near vertical plots, for all but the longest DNA sequences, emphasises that the speed up (vertical axis) comes at little reduction in quality.

**Figure 2:**
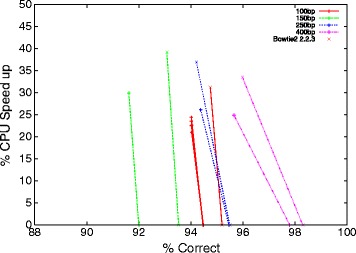
**Speed up of genetic programming versions of Bowtie2 compared to hand produced code on paired end small-indel GCAT benchmarks.** As with Table 1 and Figure 1, the percentage of correctly mapped sequences is calculated by GCAT.

In each GCAT benchmark Bowtie2 ^*GP*^ gives a speed up at the expense of a small reduction in number of correct alignments reported by GCAT, see Figures 1 and 2. Figures 1 and 2 report the percentage speed up relative to the unmodified code. That is, both Bowtie2 2.0.0 beta and Bowtie2 2.2.3 are both plotted at zero speed up.

**Table 1:** **Speed and percentage speed up of each Bowtie2 variant on GCAT benchmarks**

**Single ended**								
**Length**	**Number**	**Bowtie2 2.0.0**	**Correct**	**Mapped**	**Bowtie2** ^***GP***^	**Correct**	**Mapped**	**speedup %**
100	11945249	684 ± 6	89.23%	98.88%	782 ± 7	89.05%	98.25%	14
150	7963499	556 ± 2	93.58%	99.48%	696 ± 5	93.20%	98.58%	25
250	4778100	413 ± 9	97.14%	99.74%	509 ± 11	96.04%	98.33%	23
400	2986312	342 ± 19	98.77%	99.86%	371 ± 29	96.50%	97.69%	8
**Length**	**Number**	**Bowtie2 2.0.0**	**Correct**	**Mapped**	**Bowtie2** ^***GP***^	**Correct**	**Mapped**	**speedup %**
100	11945249	486 ± 6	93.54%	98.81%	640 ± 16	92.98%	98.16%	32
150	7963499	481 ± 3	96.33%	99.48%	701 ± 3	95.46%	98.55%	46
250	4778100	462 ± 12	98.54%	99.82%	656 ± 23	97.05%	98.40%	42
400	2986312	425 ± 41	99.36%	99.94%	524 ± 65	96.87%	97.76%	23
**Paired end**								
**Length**	**Number**	**Bowtie2 2.0.0**	**Correct**	**Mapped**	**Bowtie2** ^***GP***^	**Correct**	**Mapped**	**speedup %**
100	5972625	674	94.47%	99.41%	827	94.03%	98.70%	23
150	3981750	736	91.99%	98.82%	956	91.61%	97.62%	30
250	2389050	826	95.46%	98.96%	1041	94.38%	97.33%	26
400	1493156	658	97.79%	99.24%	822	95.65%	97.08%	25
**Length**	**Number**	**Bowtie2 2.2.3**	**Correct**	**Mapped**	**Bowtie2** ^***GP***^ **2.2.3**	**Correct**	**Mapped**	**speedup %**
100	5972625	702	95.19%	98.91%	921	94.74%	98.20%	31
150	3981750	717	93.53%	98.93%	999	93.07%	97.66%	39
250	2389050	763	95.49%	98.61%	1044	94.21%	96.83%	37
400	1493156	461	98.29%	99.39%	616	95.97%	97.24%	34

To normalise for different sequence lengths, we report millions of DNA bases processed per CPU hour. (In paired end tests both ends are counted.) Where available ± gives estimated standard deviation. The percentage of correctly assigned sequences and the percentage mapped are both reported by GCAT.

Although we do not see the spectacular speedup given by Bowtie2 ^*GP*^ on the task for which it was trained, nevertheless it does performs well on both single ended and paired end DNA sequence data. It is fair to say the original Bowtie2 was not optimised for this task, so the GP had an advantage of competing where Bowtie2 would be expected to be poor.

“Double ended” sequences require Bowtie2 to combine the results of looking up two DNA sequences (one from each end of the sequence). Naturally this combination code was not optimised when using the Broad Institute’s “single ended” data. Nevertheless both versions of Bowtie2 ^*GP*^ are able to find correct matches and retains similar speed advantages over the released versions of Bowtie2.

All the GCAT benchmarks contain sequences much longer than the 36bp single ended sequences on which Bowtie2 ^*GP*^ was optimised, nevertheless both GP versions do well on the much longer sequences. Also the speed up is similar to the 26% speed up found with real 36bp paired end sequences provided by the Cancer Institute [19], which gives some reassurance in the GCAT benchmarks. It might be expected, performance would tail away as data are less like that generated by the Broad Institute’s scanner, however speed up is fairly consistent.

With any benchmark we must be wary of over fitting. That is, getting good results on the benchmark but failing to get comparable results on real sequences. However, on the GCAT benchmarks, after two and half years of manual effort (which included rediscovering three lines of the seven line change found by genetic programming) we see an improvement in accuracy between of 0% to more than 4% (see distances between + and × on the x-axis in Figures 1 and 2). In contrast, after approximately one CPU day, genetic improvement automatically found a version with an out of sample median speed up of 27%. On the GCAT benchmark closest to the conditions it was optimised for (see 100bp in Figure 1) the reduction in accuracy is negligible.

## Discussion

There are many Bioinformatics computer based sequencing tools. In January 2013, http://en.wikipedia.org/wiki/List_of_sequence_alignment_software. Wikipedia alone listed more than 140. Each of these has been produced by hand, by some of the cleverest people on the planet and yet each can be thought of as a single point in a Pareto trade-off space in which speed, accuracy, memory requirements, etc., are balanced on different variants of the approximate string matching problem. To be successful, each author must strive to find a point in the space which is not currently dominated by an existing program. Further it should not be dominated by any future program by the time, perhaps a few years a way, when the author’s program is complete. At present, when each author starts their project, they are only guided by gut feeling and existing programs as to which points in this huge trade-off space might be reached and yet having selected a destination, few (if any) projects can fundamentally re-consider their destination if their target proves unreachable or is already occupied when they arrive. We have demonstrated GP can automatically explore around current implementations, nevertheless we hope future software designers, implementers and PhD students, will have tools which can explore potential trade-offs before implementation starts [21]. Perhaps such automatically generated software might be assembled from existing tools [22] and proved valuable, even where people choose not to adopt machine generated software but instead use it as a guide as to what is feasible before the Human starts coding.

## Conclusions

Genetic programming acting on an important program of more than fifty thousand lines of C++ code found a set of small changes which could considerably improve its performance on a task for which it was specialised. After two and a half years of manual effort three of the seven lines in the set of GP changes have been fortuitously manually incorporated into the latest release. Since GP acted on the source code, the remaining four were easily re-applied to the latest man made release.

Both GP improved versions were tested on the GCAT [20] synthetic benchmarks. They give an average speed up of 27%. This is the the same as the improvement we found on real short (36 bp) NextGen paired-end DNA sequences supplied by the Cancer Institute [19]. As judged by GCAT, for the shortest GCAT sequences we see no fall in quality, however both GP versions give approximately 2% fewer correct matches for the longest (400bp) GCAT benchmarks.

In principle, given sufficient interest, it should be possible to use the GP framework to re-optimise Bowtie2, or other sequence aligners, for tasks like those represented by GCAT.
